# Core-Shell Columns in High-Performance Liquid Chromatography: Food Analysis Applications

**DOI:** 10.1155/2016/3189724

**Published:** 2016-04-10

**Authors:** Raffaella Preti

**Affiliations:** Laboratory of Commodity Science, Department of Management, Sapienza University of Rome, Via del Castro Laurenziano 9, 00161 Rome, Italy

## Abstract

The increased separation efficiency provided by the new technology of column packed with core-shell particles in high-performance liquid chromatography (HPLC) has resulted in their widespread diffusion in several analytical fields: from pharmaceutical, biological, environmental, and toxicological. The present paper presents their most recent applications in food analysis. Their use has proved to be particularly advantageous for the determination of compounds at trace levels or when a large amount of samples must be analyzed fast using reliable and solvent-saving apparatus. The literature hereby described shows how the outstanding performances provided by core-shell particles column on a traditional HPLC instruments are comparable to those obtained with a costly UHPLC instrumentation, making this novel column a promising key tool in food analysis.

## 1. Introduction

High-performance liquid chromatography (HPLC) is an essential analytical technique for qualitative and quantitative determinations in many fields, for research, diagnostic, and manufacturing purposes. HPLC has evolved dramatically in the recent years, especially due to the development of new column technologies. In fact, in order to maximize HPLC performances in separation efficiency and speed, the size of the particles packed into the columns has been considerably decreased [1].

The traditional packed columns still dominate the market, but in the last decade columns packed with sub-2 *μ*m particles, columns packed with fully porous particles, and short columns packed with nonporous or core-shell (superficially porous) particles are gaining popularity. Sub-2 *μ*m particles columns require a dedicated instrumentation that can work at very high pressure (up to 1200–1300 bar), and therefore this technique has been called ultrahigh-performance liquid chromatography (UHPLC). By the application of this instrumentation, separations 5- to 10-fold faster than a conventional LC system can be achieved, with an important solvent saving. As a main drawback, the cost of an UHPLC apparatus is still prohibitive for an average laboratory, or it is difficult to switch from known procedures [2, 3].

The modern sub-3 *μ*m core-shell particles have a 1.7 *μ*m solid core wrapped in a porous layer or shell of a 0.5 *μ*m silica adsorbent, with a final particle size of 2.6 *μ*m. This combination of materials provides columns with speed and efficiency similar to columns packed with sub-2 *μ*m totally porous particles, while maintaining low back pressure thus can be used on conventional HPLC instrument, with a maximum pressure of 300 bar [1, 4, 5]. The porous shell provides less band broadening by reducing the dispersion of the solute molecules within a packed bed owing to a lower pore volume available for longitudinal diffusion (B term in the van Deemter equation decreased up to 30%) and a shorter diffusion path length, which diminishes the contribution of the C term to band broadening due to the fast mass transfer [5–7]. The contribution to A term (column packing regularity) is significantly reduced too (−40%), because of a rougher surface of core-shell particles [8, 9]. Also the contribution of the C term of shell particles is reduced compared to that of the fully porous particles especially for large molecules (proteins) and fast flow rates [10].

Therefore, the most important advantages achieved by the application of core-shell particles columns are the significant reduction in the time of analysis (up to 4 times less), of the reequilibration time, and, consequently, in the volume of solvents consumed. These goals are achieved without any loss in separation performance or even in an increasing of the column efficiency [8, 9].

This technology was commercialized for the first time in 2006 by Halo (Advanced Material Technologies), whose success revitalized research interest in such particle design, since the concept of superficial or shell stationary phases was introduced by Horváth et al. in the late 1960s [11].

Currently sub-3 *μ*m and sub-2 *μ*m superficially porous particles are available from several manufacturers with different length, diameter, and stationary phases (C18, C8, phenyl-hexyl, pentafluorophenyl (PFP), diphenyl, and HILIC). The most widespread brands are Accucore (Thermo Fisher Scientific), Ascentis Express (Sigma Aldrich), Cortecs (Waters), Halo (Advanced Material Technologies), Kinetex (Phenomenex), and Poroshell (Agilent Technologies). A large number of papers devoted to the analytical applications of the core-shell particles have described analytical performances comparable to those of small, fully porous particles yielding similar efficiencies. The lower backpressures allow the use of highly viscous organic/aqueous mixtures for analyzing complex matrices or analytes (biomolecules and macromolecules). Theoretical aspects and technical properties of these columns are described deeply in recent reviews [1, 10, 12, 13].

## 2. Analytical Applications

Literature describes applications of core-shell particles for quantitation of compounds of diverse analytical interest, in different matrices and with various pretreatments, making use of a variety of detectors. Most of the published applications make use of well-known pretreatment strategies, mobile phases, and detection methods. This proves how existing methods can easily be transferred to core-shell particles.

The most common applications of core-shell particles columns are in reversed-phase HPLC, allowing a reduced plate height reduction up to 1.7 [8, 9].

The analytical performances of narrow bore core-shell columns have been compared to totally porous and monolithic columns achieving better results both in efficiency and in peak asymmetry factors [12, 13]. The core-shell columns were tested also under HILIC conditions, providing fast separations and low backpressure [14, 15].

For chiral separation the results obtained were close to those of totally porous particles [16].

These innovative columns demonstrated also to be useful for improvement of analysis in comprehensive two-dimensional liquid chromatography, where they can solve the need of fast separations in the second dimension without the cost of very high operation pressure occurring when columns packed with sub-2 *μ*m particles are used. These advantages of core-shell particles columns are exhaustively reviewed by Jandera et al., 2015 [17]. Moreover, their applicability in preparative separations has been evaluated and the results showed how their use is advantageous in particular in the case of general use (e.g., purification of different compounds) in small scale separations or when it is not cost-effective to build a custom column packed with particles optimized for that given separation [18].

The main critical point in the use of these columns with a traditional HPLC is to use instruments that have much smaller contributions to band broadening than common chromatographs. A study performed by Gritti et al., 2010 [19], underlined the main improvements to an HPLC in order to ensure best column efficiency.

These columns generate peaks that occupy very small volumes. So, the contribution of instruments to the dispersion of these peaks must be reduced. The volumes of injection devices, valves, and connecting tubes, as well as that of the UV or Fluorescence Detector (FD) cell, must be minimized. Also some software adjustments should be implemented, such as the fastest sampling (40 Hz) and the minimum response time rate (0.1 s) of FD and UV, and were applied to all runs to assure that the narrowest peak was recorded completely. Therefore, very simple improvements are required to be able to take full advantage of these innovative columns. A nonoptimized HPLC will result on the contrary in an efficiency loss estimated in the range of 30–55% [19].

The advantages of core-shell particles columns, fast analysis, solvent reduction, and overall improved efficiency have been widely exploited in recent years in several research and industry fields, in particular in pharmaceutical for the determination of drugs, metabolites, and biomacromolecules [20–22], or in the analysis of proteins and peptides [23], in the analysis of environmental samples such as pesticides [4, 24, 25] or Polycyclic Aromatic Hydrocarbons (PAHs) in aqueous matrices [26]. In the present paper only recent applications in food analysis will be described.

## 3. Applications in Food Analysis

The applications of core-shell particles columns in food analysis are still at an early stage, but the articles describing the use of these columns to improve separations of several classes of compounds are getting more and more numerous in the recent years.

The present paper aim is to review the main applications of these new technology columns in the food analysis in order to highlight the results achieved and the potentialities of their use in this evolving sector. The research results are grouped according to their application area and only applications involving HPLC (not UHPLC) instrumentation will be considered.

### 3.1. Antioxidant Compounds

#### 3.1.1. Wine

Applications of core-shell particles columns in the determination of the several classes of antioxidants are numerous. Quantification of 10 phenolic acids in the wine samples was successfully performed by the use of a column packed with sub-3 *μ*m core-shell particles employed on a HPLC-UV after ion pair dispersive liquid-liquid microextraction based on the solidification of a floating organic droplet. The final results were the decreased analysis time while maintaining good separation that also required smaller volumes of solvents and smaller sample amounts compared to conventional columns without the use of a complex buffered mobile phase [27].

The optimized comprehensive two-dimensional liquid chromatography with the employment of a porous shell C18 column in the second dimension at high flow rate (4.8 mL/min) and pressure (480 bar) and elevated temperature (60°C) in combination with optimized segmented parallel gradients in the two dimensions allowed the determination of 15 flavones and 12 phenolic acids in 30 min, a time comparable to the single dimension separation [28].

*trans*-Resveratrol and* cis*-resveratrol in red wines were separated on a fused-core C18-silica column (Halo, 50 × 2.1 mm i.d., 2.7 *μ*m) and quantified by triple quadrupole linear ion trap mass spectrometry in negative ion multiple-reaction monitoring (MRM) mode after an online solid-phase extraction with multiwalled carbon nanotubes as sorbent. The proposed method achieved LOQs at a trace level (0.05 ng/mL, for* trans*-resveratrol and 0.06 ng/mL for* cis*-resveratrol) and high selectivity by a fully automated methodology, based on online SPE-HPLC-MS/MS analysis [29].

A wide variety of complex phenolic matrices have been explored, including red and white juices and wines produced from* Vitis vinifera* and interspecific hybrid grape cultivars, juices, teas, and plant extracts in the interesting article by Manns and Mansfield, 2012 [30]. These authors have optimized and validated four analytical protocols to determine the following antioxidant compounds: nonanthocyanin monomeric fraction, condensed tannins via phloroglucinolysis, and anthocyanins. The first two antioxidant classes were separated on a C18 core-shell column, while a PFP core-shell column gave better results for anthocyanins in terms of resolving power when dealing with complex anthocyanin-laden matrices such as those found in hybrid grape cultivars. The C18 and PFP core-shell columns selected for this study resulted in a dramatically improved selectivity, resolution, and throughput in each matrix and compounds tested. The protocols offered also lower solvent consumption, low detection levels, and high reproducibility using either direct injection or post-SPE injection [30].

#### 3.1.2. Cereals and Legumes

Analysis of polyphenols in cereals may be improved performing acidic hydrolysis: in a study in wheat flour and wheat bran and cereals of the diet PFP and C18 core-shell columns were compared in the determination of twelve free phenolic compounds and eight bound phenolic compounds in barley by HPLC-DAD-MS. The study of different method parameters showed that C18 column was more suitable for the analysis of phenolic compounds of barley that were separated in less than 22 min [31]. Furthermore, only the C18 column results were able to discriminate between “waxy” and “nonwaxy” barley varieties by the application of Hierarchical Cluster Analysis (HCA) [32].

Carotenoids, tocopherols, tocotrienols, alk(en)ylresorcinols, and steryl ferulates from whole grain wheat (*Triticum* spp.) flour were simultaneously quantified in 40 minutes using HPLC- DAD/FD-MS^n^ coupled with a C18 core-shell column. This column has been preliminary compared to a porous particle C30 column and to a porous particle C18 column; the core-shell column was finally selected for its higher separation power of the analytes [33].

A comprehensive characterization of phenolic compounds from the edible seeds of seven Egyptian cultivars of chickpea has been achieved by the optimization and validation of a solid-liquid extraction and of the analytical conditions of a reversed-phase- (RP-) HPLC-DAD-ESI-QTOF-MS method. The employment of a C18 core-shell particles column has led to the identification of 140 compounds, including 96 phenolic compounds, with good resolution and sensitivity. The method was suitable also for the differentiation among different cultivars [34].

Using a Kinetex PFP column, the organic concentration, gradient, and flow rate were optimized to maximize the resolution of phenolic compounds in six different subclasses of phenolic compounds in the seed coat of lentil by HPLC-MS. The Kinetex PFP has different selectivity because PFP stationary phase provides aromatic and polar selectivity by incorporating fluorine atoms on the phenyl ring; this property gives this chemistry a superior resolution and separation power for isomers compounds and hydrophilic flavones, enabling also the critical separation between luteolin-4-O-glucoside and kaempferol-3-O-glucoside in a short 30 min analysis time [35].

#### 3.1.3. Vegetables

The core-shell column 2.7 *μ*m shell particles (1.7 *μ*m nonporous core surrounded by a 0.5 *μ*m porous shell) were compared with fully porous sub-2 *μ*m particles for the separation and identification of 10 phenolic compounds in canned artichoke extracts by liquid chromatography-diode array detection-tandem mass spectrometry. The core-shell column gave the best results in terms of lower reduced plate height and flatter C term in Knox plot, together with a lower backpressure that allowed its use in a traditional HPLC with shorter analysis time and solvent consumption [36].

A method for the simultaneous determination of free chlorogenic acids and sesquiterpene lactones in chicory root and its dried (flour) and roasted (grain) forms has been described by Willeman et al., 2014 [37]. The quantitative analytic method on conventional HPLC-DAD has involved a polar reverse-phase column Kinetex PFP, chosen because of its ability to separate positional isomers. The separation performed showed a very good resolution of the target compounds in 16.5 min and no additional purification step was required as no matrix effect was demonstrated [38].

In order to be able to separate all spinach pigments in one short run a gradient separation on C18 core-shell column has been developed by Simonovska et al., 2013 [39]. All chloroplast pigments were separated: xanthophylls, including all-*trans*-violaxanthin, all-*trans*-zeaxanthin, and all-*trans*-lutein (from 1.8 min to 4.6 min), chlorophylls (7.4–9.4 min), and all-*trans*-*β*-carotene (12.4 min). Two neoxanthins were well separated from the all-*trans-*violaxanthin. All-*trans*-lutein and all-*trans*-zeaxanthin peaks presented poor resolution. Since the low content of zeaxanthin in spinach, this drawback was tolerable. To have a better resolution of lutein and its structural isomer zeaxanthin, important carotenoids in the eye retina, a longer analysis using a C30 column was optimized [39].

A rapid analytical method for extraction, characterization, and quantification of the major polyphenolic constituents in* Salvia* species has been developed for a fast screening and preliminary quality assessment. For fast separation methodology, the selectivity and efficiency of three different reversed-phase C18 stationary phases: extra-density bonded (XDB), core-shell technology, and monolith column, were compared to a traditional stationary phase column using LC–DAD–ESI-MS^n^. The method developed using the core-shell technology produced satisfactory resolution of all components of interest in an acceptable run time (20 min), even if on the traditional column five times more compounds could be identified in a longer time of 90 min [40].

#### 3.1.4. Fruits

The fruit of wild* Berberis* species of South America have shown a high antioxidant capacity and a very high content in anthocyanins and hydroxycinnamic acid derivatives that have highlighted its potentiality as nutraceutical food product. To complete the knowledge of the functional properties of these berries, a study on their flavonols profile and content was carried out by HPLC-DAD. To perform this analysis with best efficiency three reversed-phase C18 columns were evaluated: a traditional HPLC column (250 × 4.6 mm, 5 *μ*m), a core-shell column, and an UHPLC column, with core-shell column giving the best results [41].

The determination of the important antioxidant class of thiols in fresh fruit samples (watermelon, melon, and avocado) was carried out by HPLC/UV on a C18 (50 × 2.1 mm i.d., 2.7 *μ*m) fused-core column, using methyl propiolate as an advantageous precolumn derivatization reagent. The method proposed, with a total analysis time of less than 10 min, low limits of detection, and satisfactory linearity for all analytes, proved to be suitable for green chemistry determinations [42].

### 3.2. Triacylglycerols

In order to obtain TAGs separation in vegetable oils with optimal resolution within a short analysis time, the use of a C18 core-shell particles column was tried with both UV and ESI-Q-ToF tandem mass spectrometer detection [43]. The two methods showed an improvement of plate height and overall efficiency, being the first approach also successfully applied to the identification of EVOO adulteration with other low cost oils by Linear Discriminant Analysis (LDA) and therefore proposed as a convenient oil adulteration screening method [44].

TAGs profile is also important for food of animal origin, and the possibility of classifying milks according to their mammalian origin by using this parameter obtained by HPLC-ELSD has been explored by Ten-Doménech and coauthors and a high throughput method employing a C18 core-shell column was developed [45].

### 3.3. Food Contaminants

#### 3.3.1. Bisphenols

Bisphenol A diglycidyl ether, bisphenol F diglycidyl ether, and their hydrolyzed derivatives are common at the base for the production of coatings for the inside of food containers. They can migrate into the packed foods and for their toxicity their presence is limited by EU legislation. A fast LC-MS/MS method for their analysis was validated in canned food samples and soft-drink beverages after a simple sample treatment procedure by the application of a C18 porous shell particle column, achieving a total run time of less than 5 min [38].

The same research group applied this column also to the simultaneous analysis of bisphenol A (BPA), bisphenol F (BPF), bisphenol E (BPE), bisphenol B (BPB), and bisphenol S (BPS) in canned soft drinks without any sample pretreatment. The analysis of bisphenols in soft drinks has been carried out by online solid-phase extraction fast liquid chromatography-tandem mass spectrometry [46].

#### 3.3.2. Natural Contaminants

Natural contaminants are toxic compounds produced naturally by several microorganisms, their concentration is influenced by several factors, and often their presence in trace concentrations is unavoidable.

*(1) Mycotoxins.* Mycotoxins are low molecular compounds, produced by the secondary metabolism of fungi. They can exert toxic and/or carcinogenetic actions in animals and humans, in some cases even at low concentrations close to 1 *μ*g/kg levels.

Ochratoxin A (OTA) is often present in red wines, where its EU legal limit is 2 *μ*g/L. Being a mandatory limit, its fast analysis is of particular importance due to the huge number of samples to be routinely controlled. In this contest, the fast quantitative method based on a core-shell particle column for Ochratoxin A by HPLC/FD would permit large savings without hardware update or modification. In fact, the transfer of analytical methods from the old to the new column can ensure efficient, sensitive, and accurate quantification of OTA with an outstanding sample throughout and a run time of less than 5 min [3]. The same approach has been applied to the determination of Aflatoxin M1, a carcinogenic compound occurring in milk, in which EU limit is set at 0.05 mg/kg for adult consumption and was set more restrictively to 0.025 mg/kg for infants and young children, baby food products. This method has been optimized by the application of a large volume injection (100 *μ*L) and stepwise gradient elution that together with a sensitivity improvement contributed to fulfil strict legal requirements avoiding any solvent replacement [47].

The presence of mycotoxins is reported also in agricultural products; a new quantitative method has been described for the determination of five* Alternaria* toxins and citrinin in tomato and tomato juice samples based on LC-MS/MS detection and core-shell column [48].

Microcystins are natural contaminant produced by cyanobacteria. Because of their high toxicities, the World Health Organization (WHO) has established an allowable level of 1 g/L in drinking water. Hayama et al. have recently proposed a separation on a PFP core-shell column and fluorescence detection after precolumn excimer fluorescence derivatization with 4-(1-pyrene) butanoic acid hydrazide. The method achieved good sensitivity, allowing to quantify a tenth of the maximum allowable value (0.1 g/L) of microcystins in drinking water [49].

The mycotoxin most frequently detected in cereal products is deoxynivalenol (DON). Recently, researchers and authorities pay attention also to its metabolites due to the effects of plant metabolism and technological processes. The main DON metabolite is the 3-*β*-D-glucopyranosyl-4-deoxynivalenol (DON-3G), whose determination is therefore important for a correct estimation of DON presence in cereal products. The method proposed by Suman et al. solved this task involving a core-shell particle column coupled with liquid chromatography/linear ion trap mass spectrometry [50].

*(2) Biogenic Amines.* The determination of biogenic amines is important for food safety but they are also considered a food quality index [51]. Their HPLC determination usually requires a derivatization step, due to the absence of chromophore or fluorescent groups, and their chromatographic separation in wine has recently been improved by the use of a PFP core-shell technology [52]. This study describes the determination of the main 4 biogenic amines present in wine (histamine, tyramine, putrescine, and cadaverine) in 25 minutes, while demonstrating the separation of the other amines compared to histamine in 10 minutes.

Recently, biogenic amines have also been employed in wine authentication [53–56]. In this contest, the analysis of a wider number of biogenic amines is useful to highlight all the significant differences and achieve a good differentiation among the wine categories. For this purpose, our research group has developed a new method for the separation of 11 biogenic amines in wine and fruit nectars by the application of a C18 core-shell column on a traditional HPLC, which in comparison to the previous reported analytical methods showed a higher sensitivity and a reduction of the run time by 60% and reduction of the solvent use by 75%. These results are comparable to those achieved by the use of sub-2 *μ*m particles column (Table 1).

The method has been validated both on UV and on FD detectors, to satisfy the needs of the average quality control laboratory [54]. This optimized method applied to the determination of biogenic amines in 60 Italian wines allowed the classification of the samples according to their retail price together with their antioxidant properties [55].

In another study the correct classification of 56 wines of Southern Italy was allowed according to their protected designation of origin (PDO) by chemometric techniques [56]. The method has been modified in order to be applied to the analysis of biogenic amines in concentrated fruit juices, a matrix not yet explored from this toxicological aspect.

Recently, alkaloids quantification in food has gained attention due to their liver toxicity. Herbal teas have been shown to contain pyrrolizidine alkaloids (PA), one of the most dangerous compounds of this class. Therefore, the fats and reliable determination by LC-ESI-MS/MS and a PFP core-shell column developed in this matrix [57] are an important starting point for the safety assessment of herbal teas and also for traditional Chinese medicines.

The presence of alkaloids and berberine in particular was assessed in edible wild* Berberis* species from Patagonia which was detected only in seeds by the application of a C18 core-shell column and at a level not relevant from a toxicological point of view [41].

The supplanting of the original fully porous particle column with a core-shell particle column in the AOAC Official Method 2005.06^SM^ for the analysis of paralytic shellfish toxins in seafood resulted in the reduction of 66% in sample analysis time [58, 59].

#### 3.3.3. Antibiotics Residues in Food

The presence of antibiotics residues in food for human consumption is a well-known food safety issue. In fact, antibiotics used as veterinary drugs in cattle often are present at dangerous levels in the animal origin food, creating toxicological problems or resistance phenomena [60].

The application of modern core-shell column in the analysis of antibiotic residues can offer many advantages, such as the reduction of analysis time and LODs. There are many recent methods developed in this direction. Tolgyesi et al., 2012 [61], demonstrated that the use of a C18 core-shell particle column in the detection of lincomycin in honey, muscle, milk, and eggs by LC-MS-MS resulted in an analysis time and in LODs at least three times lower than the previous methods.

By the use of a C18 core-shell particle column, also the levels of tetracyclines in pig, bovine, chicken, and turkey meat samples [62] and of the five currently utilized polypeptide antibiotics in muscle, liver, kidney, egg, and milk [63] were successfully determined both in HPLC-DAD and HPLC-MS/MS. A fast and reliable determination to control the illegal use of the nine regulated quinolones in eggs was achieved in HPLC/FD [64].

Separation with the newly developed Kinetex C18 core-shell technology column resulted in a significant reduction in solvent consumption and in analysis time, combined with good resolution for all analytes and better sensitivity without the need of costly MS instrumentation, even if this technique offers the possibility of an analysis time of 5 min ensuring higher selectivity and sensitivity, when applied to complex matrices such as food of animal origin [65].

## 4. Conclusions

In the last decade, the novel core-shell particles columns technology has gained a position of primary importance in liquid chromatography. Their popularity is due to many advantages such as speed of analysis and resolution power that can be achieved on a common HPLC apparatus with minimal adjustments, thanks to the low backpressure guaranteed by these columns. Their application can therefore allow reaching analytical performances of sub-2 *μ*m particles columns without affording the cost of an UHPLC instrumentation.

Also the research for greener analytical methods can find in these columns a satisfactory and reliable answer, due to the dramatic reduction in solvent consumption and in waste production.

The columns packed with 10 and 5 *μ*m fully porous particles dominated the field for nearly thirty years, but nowadays their use shows less benefits compared to core-shell particles columns not only in terms of height equivalent to a theoretical plate, but also in overall kinetic performance.

The literature hereby described in food analysis demonstrates how this approach can significantly improve this emerging analytical task. In food analysis, core-shell particles columns can really represent a keynote technology for several reasons: in consideration that legal maximum limits permitted for many contaminants are often at trace level; the huge amount of samples required for control and authentication purposes; the instrumentation cost, affordable also for average laboratories in developing countries.

Therefore, the continuous evaluation and investigation of core-shell particles in the development of highly efficient separation methods applied to relevant compounds in complex food matrices is highly expected and encouraged by the scientific community.

## Figures and Tables

**Table 1:** Literature comparison to the proposed method for the determination of biogenic amines (adapted from [54]).

Apparatus, derivatization agent	Biogenic amines	Analytical column, flow	Run time (min)	Time per analyte (min)	Solvent consumption (mL)	Matrix	Ref.
HPLC-FD Dansyl-Cl	MEA, ETA, AGM, *β*-PEA, PUT, CAD, HIS, IS, SER, TYM, SPD, SPM	Kinetex C18 100 × 4.6 mm 2.6 *µ*m Φ = 0.6 mL/min	13	1.1	7.8	Wine, fruit nectar	[54]

HPLC-FD AccQ-Fluor*™* Reagent Kit	PUT, CAD, HIS, IS, TYM	Kinetex PFP 100 × 4.6 mm 2.6 *μ*m Φ = 0.8 mL/min	25	5	20	Wine	[52]

HPLC-UV Dansyl-Cl	AGM, *β*-PEA, PUT, CAD, HIS, IS, TRYP, TYM, SPD, SPM	Chrompack Hypersil C18 250 × 3.0 mm 5 *µ*m Φ = 1 mL/min	29	2.9	29	Fish	[66]

HPLC-UV Dansyl-Cl	CAD, PUT, HIS, TYR, TRYP, PHE, IS, SPD, SPM, MEA, ETA	C18 Supelco Discovery 150 × 2.1 mm 5 *µ*m Φ = 0.4 mL/min	30	2.7	12	Wine	[67]

HPLC-FD Dansyl-Cl	CAD, PUT, HIS, TYR, TRYP, AGM, ISO, MEA, ETA	C18. Luna 250 × 4.6 mm 5 *µ*m Φ = 1 mL/min	35	3.9	35	Wine	[68]

Ion pair HPLC OPA	SPD, SPM, PUT, AGM, SYN, TYM, OCT, HIS, SER, CAD, TRYP, *β*-PEA	Bondapak C18 300 × 3.9 mm 10 *µ*m Φ = 0.7 mL/min	71	5.9	50	Orange juice	[69]

HPLC-UV Dansyl-Cl	AGM, *β*-PEA, PUT, CAD, HIS, IS, TRYP, TYM, SPD, SPM	Zorbax Eclipse C18 50 × 4.6 mm 1.8 *µ*m Φ = 1 mL/min	12	1.2	12	Fish	[70]

UHPLC-UV Dansyl-Cl	TRYP, *β*-PEA, PUT, CAD, HIS, IS, TYM, SPD, SPM	Zorbax Eclipse C18 50 × 4.6 mm 1.8 *µ*m Φ = 1 mL/min	12	1.3	12	Meat, cheese, mushrooms	[71]

UHPLC FD OPA	OC, DO, TYR, PUT, SER, CAD, HIS, AGM, *β*-PEA, SPD, TRYP, SPM	Acquity UPLC BEH 50 × 4.6 mm 1.7 *µ*m Φ = 0.8 mL/min	7	0.6	5.6	Wine, fish, cheese, dry sausages	[57]

OPA, o-phthalaldehyde; SPD, spermidine; SPM, spermine; PUT, putrescine; AGM, agmatine; SYN, synephrine; TYM, tyramine; OCT, octopamine; CAD, cadaverine; HIS, histamine; SER, serotonin; TRYP, tryptamine; ETA, ethylamine; MEA, methylamine; HIS, histamine; *β*-PEA, *β*-phenylethylamine; DO, dopamine; ISO, isopropylamine; IS, internal standard.
